# N-Doped Carbon Fibers Derived from Porous Wood Fibers Encapsulated in a Zeolitic Imidazolate Framework as an Electrode Material for Supercapacitors

**DOI:** 10.3390/molecules28073081

**Published:** 2023-03-30

**Authors:** Zhen Zhang, Yan Qing, Delong Wang, Lei Li, Yiqiang Wu

**Affiliations:** 1 Hunan Provincial Collaborative Innovation Center for High-Efficiency Utilization of Wood and Bamboo Resources, College of Materials Science and Engineering, Central South University of Forestry and Technology, Changsha 410004, China; 2 Forestry Engineering, Northeast Forestry University, Harbin 150040, China; 3 Datang Hubei New Energy Division, Huanggang 438000, China

**Keywords:** zeolite imidazole framework, N-doping, wood fibers, carbon electrodes, supercapacitors

## Abstract

Developing highly porous and conductive carbon electrodes is crucial for high-performance electrochemical double-layer capacitors. We provide a method for preparing supercapacitor electrode materials using zeolitic imidazolate framework-8 (ZIF-8)-coated wood fibers. The material has high nitrogen (N)-doping content and a specific surface area of 593.52 m^2^ g^−1^. When used as a supercapacitor electrode, the composite exhibits a high specific capacitance of 270.74 F g^−1^, with an excellent capacitance retention rate of 98.4% after 10,000 cycles. The symmetrical supercapacitors (SSCs) with two carbon fiber electrodes (CWFZ2) showed a high power density of 2272.73 W kg^−1^ (at an energy density of 2.46 W h kg^−1^) and an energy density of 4.15 Wh kg^−1^ (at a power density of 113.64 W kg^−1^). Moreover, the SSCs maintained 81.21% of the initial capacitance after 10,000 cycles at a current density of 10 A g^−1^, which proves that the SSCs have good cycle stability. The excellent capacitance performance is primarily attributed to the high conductivity and N source provided by the zeolite imidazole framework. Because of this carbon material’s unique structural features and N-doping, our obtained CWFZ2 electrode material could be a candidate for high-performance supercapacitor electrode materials.

## 1. Introduction

In the past few decades, the shortage of fossil energy and the deteriorating environment have affected society’s sustainable development. Developing green, low-cost, and sustainable energy storage materials and devices is critical [1,2,3]. Among the many energy storage devices, supercapacitors (SCs) have received extensive attention from researchers because of their fast charge–discharge rates and excellent cycle stability. In addition, SCs can compensate for the shortcomings of the low energy density of traditional capacitors and low power of density of batteries, meeting the application requirements of energy storage systems, such as backup power supplies and electric vehicles [4,5,6]. Their energy storage mechanism divides SCs into pseudocapacitors and electrochemical double-layer capacitors (EDLCs). Pseudocapacitors chemically store charges through surface oxidation-reduction reactions. However, EDLCs store energy by accumulating charges on the electrodes without electrochemical reactions [7,8]; therefore, they have excellent power density and electrochemical stability.

The typically used electrodes in EDLCs are carbon-based because of their high specific surface area (SSA), good electrical conductivity, and nontoxic and harmless characteristics [9,10,11,12]. In recent years, as a new type of porous crystalline metal-organic framework (MOF), the zeolite imidazole framework (ZIF) has become the most popular porous carbon (PC) precursor or sacrificial template [13,14]. Because of its flexible pore structure, stable chemical properties, and easy synthesis, ZIF-8 is mostly used to prepare electrodes among ZIF materials. Chaikittisilp prepared nanoPC via the high-temperature carbonization of ZIF-8 as an SC electrode (ZIF-800), exhibiting a specific capacitance of 130 F g^−1^ at a scan rate of 50 mV s^−1^ [15]. Gao studied the carbon-based electrodes obtained from carbonizing ZIF-8 under various atmospheric conditions and found that ZIF-8-derived carbon materials possess the most significant specific capacitance under nitrogen protection at a scan rate of 5 mV s^−1^ [16]. Wu prepared nanohexahedral PC (PC-850) by directly carbonizing ZIF-8, obtaining an SSA as high as 1142 m^2^ g^2^ and excellent cycle stability [17]. However, the PC material directly obtained from carbonizing ZIF-8 comprises dispersed nanoparticles with a low degree of graphitization and easy agglomeration. This morphological structure limits the ion conduction from the electrolyte to the electrode material, causing low conductivity and poor rate performance in the electrode material [18,19].

Therefore, to solve these problems, the current strategy is to increase the secondary carbon source to improve the electrode material’s conductivity, such as carbon nanotubes (CNTs), graphene, carbon aerogels, and conductive polymers. Wang used graphene functionalized with polyetherimide as the base material to anchor the ZIF-8-obtained carbon frameworks, exhibiting a specific capacitance of 218 F g^−1^ at a current density of 0.5 A g^−1^ and a good rate performance [20]. Wang obtained nanocomposite carbon materials (PC/CNTs) by pyrolyzing polypyrrole nanotubes (PNTs) coated with ZIF-8. PC/CNTs were prepared into an SC electrode, delivering a specific capacitance of 250 F g^−1^ at a current density of 0.1 A g^−1^ and a low resistance (0.53 Ω) [21]. Zhang used electrochemical deposition to load ZIF-8 on oxidized multiwalled CNTs to prepare a necklace-like composite material. The PC material (C-ZIF-8@MWCNTs), which was obtained after carbonization and acid treatment, showed high conductivity and a specific capacitance of 259.2 F g^−1^ [22]. However, most carbon materials, such as CNT and graphene, need to be activated and modified before use, which increases the cost, and the preparation process is complicated. Therefore, it is crucial to find other secondary carbon sources to replace CNT and graphene.

Wood fiber (WF) is a kind of natural and renewable polymer material that is normally used in traditional fields such as wood-based panel manufacturing, pulp and paper making, etc. With the development and progress of technology, wood fiber, a material with high carbon content and abundant surface active functional-groups, has been used to prepare carbon-based electrode materials. Inspired by the previous work, we propose a strategy using WF to synthesize concentric shaft-coated structural carbon materials without surface modifiers as electrode materials. The chemical composition of WF is mainly cellulose, hemicellulose, and lignin. The surface is exposed to a large number of active functional groups, such as the hydroxyl and carboxyl groups, which play a crucial role in fixing active substances. Therefore, WF can fix ZIF-8 well and prevent its accumulation during the synthesis process, and also provide a secondary carbon source with excellent conductivity. Simultaneously, ZIF-8 provides a source for N doping for carbon materials and improves its carbon structure, specific surface area, and conductivity. The results show that the obtained carbon fiber (CWFZ2) electrode material exhibits high energy storage performance (specific capacitance of 270.74 F g^−1^ at a current density of 0.5 A g^−1^) and excellent cycle stability (retaining 98.4% of the initial capacitance after 10,000 cycles of cyclic voltammetry). As an electrode material, the obtained CWFZ2 is incorporated into a symmetrical SC (SSC) device exhibiting a high power density of 2272.73 W kg^−1^ and an energy density of 4.15 Wh kg^−1^. Accordingly, the CWFZ2 has strong application potential as an electrode material in SCs.

## 2. Results and Discussion

### 2.1. The Morphology and Structure of Different Samples

**Figure 1:**
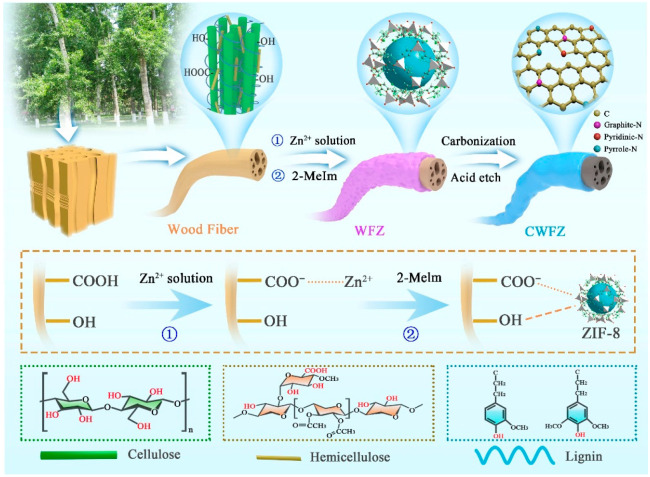
Schematic representation of CWFZs synthesis.

**Figure 2:**
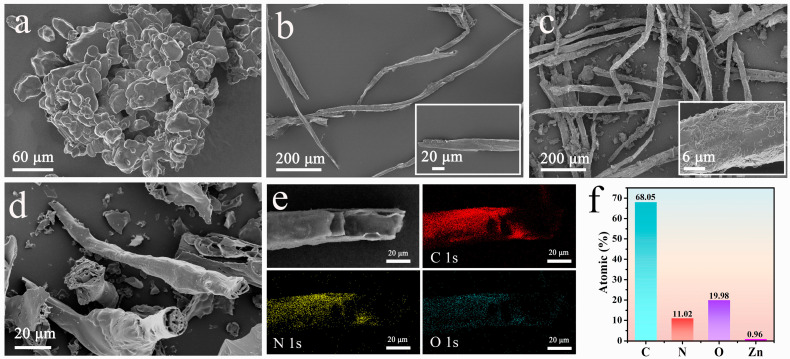
SEM images of (**a**) ZIF-8, (**b**) WF, (**c**) WFZ, and (**d**) CWFZ2; (**e**) STEM and element mapping of CWFZ2; (**f**) Different atomic content of CWFZ2.

Figure 1 is a schematic diagram of the preparation of CWFZs. While ZIF-8 provides a porous network, WF, with a large number of active functional groups on the surface, is used as a mechanical support to build a new type of composite material. In the first step, WF with a relatively smooth surface (Figure 2b) was immersed in an aqueous solution containing zinc nitrate, and the abundant hydroxyl and carboxyl groups on the WF’ surface anchored the zinc ions through electrostatic adsorption. Subsequently, the WFs loaded with zinc ions were added to the dimethylimidazole solution for in situ growth, and the ZIF-8 coating layer was formed by heterogeneous nucleation on the WF’s surface. Figure 2c shows that ZIF-8 is uniformly dispersed on the fiber surface, effectively avoiding the agglomeration of ZIF-8 nanoparticles (Figure 2a). The core-shell carbon fibers (Figure 2d) were obtained from high-temperature calcination, and ZIF-8 was pyrolyzed into N-doped carbon. More detailed synthetic procedures are provided in the Methods section. Furthermore, as shown in Figure 2e, the energy dispersive spectroscopy (EDS) mapping analysis indicated that the C, N, and O elements were uniformly distributed on the WF’ surface. Figure 2f shows the element content of CWFZ2. The trace amount of remaining Zn atoms in it can be ignored. A high content of N-doping can promote the electrochemical performance of carbon electrode materials.

**Figure 3:**
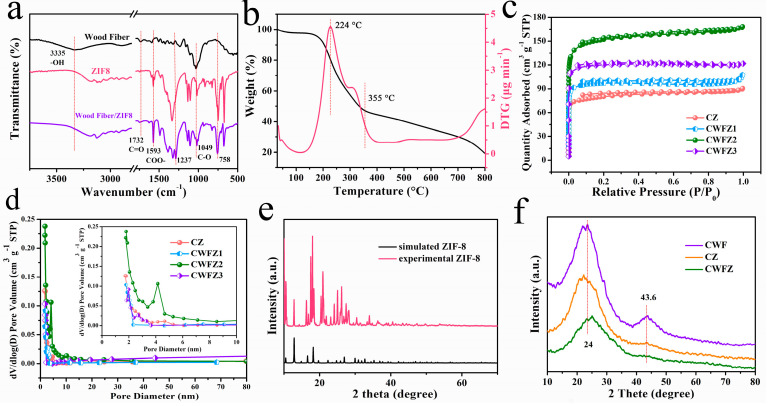
(**a**) FTIR patterns of WF, ZIF-8, and WFZ; (**b**) TG and DTG curves of WFZ; (**c**) N_2_ adsorption-desorption isotherm and (**d**) the pore size distribution of CZ and CWFZ2; (**e**,**f**) XRD patterns of different samples.

Figure 3a shows the FTIR of WF, ZIF-8, and WF@ZIF-8, confirming the change in functional groups. A unique band with a peak at 3335 cm^−1^ indicates the –OH functional group of WF. The peak at 1237 cm^−1^ is due to the uronic acid groups in hemicellulose or the ester bonds between the lignin and hemicellulose carboxyl groups. A decrease in carbonyl intensity (–C=O) at 1732 cm^−1^ and an increase in carboxyl intensity (COO–) at 1593 cm^−1^ indicate that some carbonyl groups are bound to ZIF-8 in situ. Furthermore, a unique band at 1049 cm^−1^ indicates cellulose has C–O–C or C–O vibrations. The FTIR spectrum of WF@ZIF-8 shows a band at 758 cm^−1^, which is related to the out-of-plane bending of the 2-methylimidazole (2-MeIm) ring of ZIF-8 [23,24]. The WF and WFZ calcining were characterized by the thermogravimetric and the thermogravimetric derivative. Figure 3b shows that the mass loss of the WFZ occurs first at ~128 °C, which is mainly caused by the loss of water in the sample. And the most rapid mass-loss rate of WF and WFZ was observed at 224 °C, caused by the decomposing compound in 2-MeIm. The degradation of cellulose and hemicellulose causes the mass loss of WF at 233 °C. When the carbonization temperature reaches 355 °C, the fastest loss of WF quality is due to the degradation of cellulose, hemicellulose, and lignin.

Figure 3c shows the N_2_ adsorption–desorption isotherms of carbonized ZIF-8 (CZ) and different CWFZs. The adsorption–desorption curves of CZ and different CWFZs overlap, and the isotherms increase sharply at low relative pressures (P/P_0_ < 0.01), indicating the existence of microporous structures inside all samples. Simultaneously, CZ and the different CWFZs samples have a hysteresis loop under moderate relative pressure (0.3 < P/P_0_ < 0.9), indicating a mesoporous structure in the sample [25]. Because of its hierarchical porous structure, the SSA of CZ, CWFZ1, CWFZ2, and CWFZ3 is 313.03, 342.16, 593.52, and 415.74 m^2^/g, respectively. Furthermore, the pore size distribution map proves that CZ and the different CWFZs have hierarchical porous structures with micropores and mesopores (Figure 3d). The primary apertures of CZ and the different CWFZs are about 4.16 nm, and the pore volume of CZ, CWFZ1, CWFZ2, and CWFZ3 is 0.139, 0.161, 0.260, and 0.194 cm^3^/g, respectively. This hierarchical porosity structure, high SSA, and large pore volume provide a high electroactive area and accelerate the diffusion of electrolyte ions, enhancing the capacitance and rate capability of the electrode [26]. This result is due to the uniform dispersion of WFs in ZIF8.

**Figure 4:**
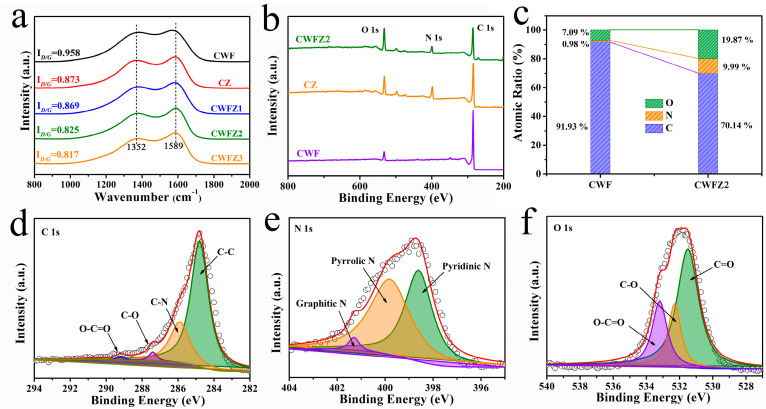
(**a**) Raman spectra of different samples; (**b**) XPS survey spectrum and (**c**) atomic content of different samples; (**d**–**f**) high-resolution spectra of C 1s, N 1s, and O 1s.

The crystallographic structures of the different samples were characterized using XRD measurements. The spectrum of the ZIF-8 synthesized in this experiment correlates with the simulated crystal structure spectrum previously published in Figure 3e. The diffraction peaks of ZIF8 appeared at 7.5°, 10.5°, 12.7°, 14.8°, 16.8°, 18.1°, and 19.3°. After the calcinations (Figure 3f), all the diffraction peaks of ZIF-8 disappeared, and two characteristic peaks appeared at 24.0° and 43.6°, corresponding to (002) and (100) crystallographic planes of disordered and amorphous carbon, indicating that the ZIF-8 nanoparticles were transformed into carbon nanoparticles [27,28,29]. No diffraction peaks for Zn impurities and other diffraction peaks for ZIF-8 are observed in the PCs. In order to better study the properties of the carbon materials, Raman tests were conducted on the different samples (Figure 4a). Two peaks appeared at 1352 cm^−1^ and 1589 cm^−1^ in all the samples, corresponding to defect regions (D-band) and ordered carbon (G-band), respectively. Overall, the intensity ratio of the D-band to the G-band (I_D_/I_G_) can confirm the defect density of carbon [30]. It can be seen that the I_D_/I_G_ values of CWFZs are lower than CWF (0.958) and CZ (0.873), indicating that there are fewer disordered regions, which is beneficial for energy storage [31].

The surface chemical compositions of the samples were further investigated using XPS measurements plotted in Figure 4b. The C, N, and O signals are at binding energies of ~285.8, 399.7, and 532.1 eV, respectively [32,33]. The C, N, and O content analyses in CWF and CWFZ are summarized in Figure 4c, which is almost consistent with the result of the EDS test. CWFZ has a higher heteroatom content (N and O at 29.86%) due to the heteroatom-doping effect of ZIF-8 on the material. As shown in Figure 4d, the XPS spectrum of C 1s can be deconvoluted into four fitting peaks. The main peak at 284.7 eV corresponds to the C–C group and belongs to the *sp*^2^ hybrid graphite-like carbon structure, which means that most of the C atoms are arranged in the conjugate honeycomb lattice. The peak at 285.9 eV is related to the N-*sp*^2^ C, which also confirms that nitrogen doping is occurring in the carbon skeleton. The peaks at 287.3 eV and 288.8 eV correspond to the C–O bond and O–C=O bond, respectively. From the results, it is further confirmed that N bonding to the carbon skeleton has a potential contribution to its electrochemical performance [34,35]. The high-resolution N 1s spectrum can be fitted into three peaks (Figure 4e). The peaks located at 397.9 eV, 399.5 eV, and 401.1 eV represent pyridine N (N-6), pyrrolic N (N-5), and graphitic nitrogen (N-Q), respectively [36]. These peaks confirmed the fact that N was doped into the CWFZ2 material. The above studies prove that N forms N-6 by replacing the C atoms on the defect sites in the graphitic carbon layer. The high chemical activity and low energy barrier of N-6 and N-5 can induce defects at electrochemically active sites, enhancing the charge storage capacity of carbon materials [37]. N-Q can enhance the electron transfer of carbon materials [38]. All these groups are conducive to obtaining excellent SC electrode materials. The results show that N atoms are doped into the carbon structure, which is consistent with the C 1s spectrum. Moreover, in the O 1s XPS spectrum (Figure 4f), the deconvolution of the O 1s peak provides three primary peaks at 531.5, 532.3, and 533.6 eV, representing the C=O, C–O, and O–C=O bonds, respectively. These oxygen-containing groups might provide additional pseudocapacitance for SCs [39,40]. Heteroatom groups (including nitrogen- and oxygen-containing functional groups) doped into carbon frameworks can enhance the electrical conductivity of carbon materials [41].

### 2.2. The Electrochemical Performances of Different Samples

**Figure 5:**
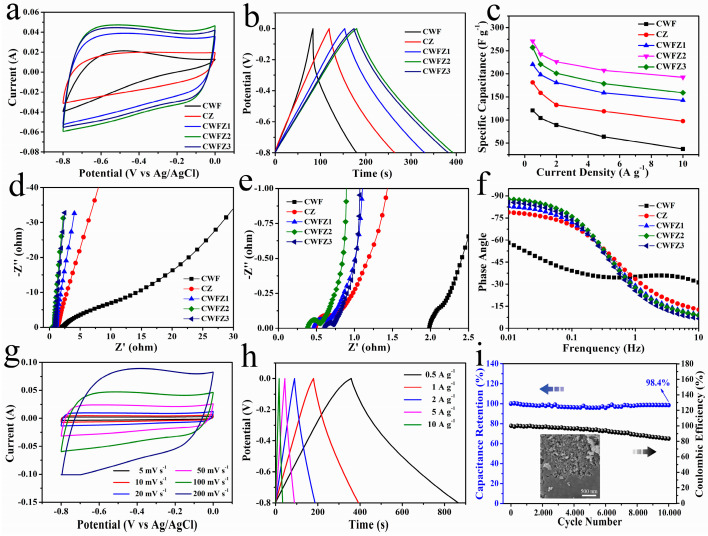
(**a**) CV curves of different electrodes at a scan rate of 100 mV s^−1^; (**b**) GCD curves of different electrodes at a current density of 5 mA cm^−2^; (**c**) specific capacitance of different electrodes; (**d**,**e**) EIS curves of different electrodes; (**f**) Bode phase diagrams of different electrodes; (**g**) CV curves of CWFZ2 at different scan rates; (**h**) GCD curves of CWFZ2 at different current density; (**i**) cycle stability and a Coulomb efficiency of CWFZ2, insert map shows the SEM of CWFZ2 after electrochemical testing.

The electrochemical performance of the samples was evaluated using a three-electrode system in 6 M KOH. The graph shows the sample’s CV curve at 100 mV s^−1^ in a voltage window from −0.08 to 0 V (Figure 5a). The CV curve area of CWFZ2 is the largest, indicating that it has the most prominent specific capacitance. The CV curve areas of the different electrodes exhibit quasirectangularity, indicating that they have exemplary electrochemical capacitive behavior and high-rate capabilities [42]. Figure 5b shows various electrodes’ galvanostatic charge–discharge (GCD) curves. According to Formula (1), the specific capacitances of CWF, CZ, CWFZ1, CWFZ2, and CWFZ3 at a current density of 1 A g^−1^ can be calculated as 104.3, 158.8, 198.0, 242.0, and 220.6 F g^−1^, respectively. Therefore, the synergistic effect of WF and ZIF-8 provides the CWFZ2 with excellent capacitive performance. Figure 5c shows the specific capacitance of the samples at various current densities. CWFZ2 maintains a specific capacitance of 192.5 F g^−1^ at a high discharge rate of 10 A g^−1^. Its excellent rate capability performance rate can be attributed to the N self-doping of carbon materials by ZIF-8, good wetting properties, and low resistance.

Figure 5d,e show the Nyquist plot of the sample from 100 kHz to 0.01 Hz. The samples exhibited a typical semicircular arc in the high-frequency region and an almost vertical line at low frequencies, demonstrating a high capacitance behavior consistent with the CV results [12]. The Nyquist intercept on the *x*-axis (0.38 Ω) of CWFZ2 is shorter than that of CWF (1.98 Ω), CZ (0.51 Ω), CWFZ1 (0.47 Ω), and CWFZ3 (0.61 Ω). Therefore, the equivalent series resistance (ESR) of CWFZ2 is the lowest, including electrode resistance, the contact resistance between the electrode and the current collector, and the ionic resistance of the electrolyte [43]. The straight line of CWFZ2 in the high-frequency region is highly parallel to the *y*-axis, indicating its ideal EDLC behavior and fast ion diffusion and transfer. The extremely low electrical resistance of CWFZ2 is primarily due to the excellent conductive base provided by the fibers, the partial graphitization of the carbon materials, and the hierarchical porous structure. The microporous and mesoporous structures in the sample can provide large spaces for ion diffusion, which can then improve the rate ability. From the Bode phase diagram in Figure 5f, the phase angles of CWZ1, CWZ2, and CWZ3 are 82.4°, 88.5°, and 85.2°, respectively, which is close to the 90° phase angle of an ideal capacitor [44].

The CV curve shapes (Figure 5g) of CWFZ2 can still maintain a good rectangular shape at scan rates of 5–200 mV s^−1^, indicating good charge transfer characteristics. Figure 5h shows that the GCD curves of CWFZ2 exhibit an ideal symmetrical triangle, indicating excellent characteristics of storing charges in the electric double layer. Specifically, the capacitance of CWFZ2 was ~270.74 F g^−1^ at a current density of 0.5 A g^−1^, which is significantly higher than that of the other carbon-based electrode materials previously reported, such as PC-850 (132.8 F g^−1^ at 0.5 A g^−1^) [17], GPNC (218 F g^−1^ at 0.5 A g^−1^) [20], N-HPC (128.5 F g^−1^ at 0.2 A g^−1^) [45], CMCMs (210 F g^−1^ at 1 A g^−1^) [46], H-N-N-*T* (162 F g^−1^ at 1.25 A g^−1^) [47], and PC1000@C (225 F g^−1^ at 0.5 A g^−1^) [48]. The electrochemical stability of CWFZ2 was evaluated by repeating the GCD at a current density of 2 A g^−1^ (Figure 5i). The specific capacitance of CWFZ2 can maintain 98.4% of the initial specific capacitance after 10,000 cycles, demonstrating its superior cyclic charge–discharge stability and good reversibility. The Coulomb efficiency is at a high level during the whole cycle, indicating high energy utilization efficiency. This result could be due to the balance of the high surface area, high N content, and high electrical conductivity of CWFZ2 [49]. After electrochemical testing, more pore structures appear on the surface of the electrode material, which can be used as ion buffers to provide short diffusion distances and the stable transport of electrolyte ions into the bulk material. This is also the reason why the specific capacitance of the electrode material has increased during the cyclic testing process.

**Figure 6:**
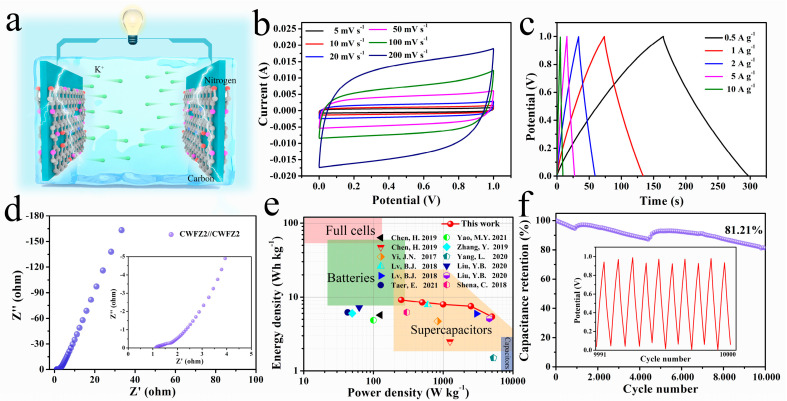
(**a**) Schematic diagram of SSCs; (**b**–**d**) CV, GCD, and EIS curves of SSCs; (**e**) Ragone plot of SSCs; (**f**) cycling performance of SSCs for 10,000 cycles and the last 10-cycle GCD curves of the SSCs [51,52,53,54,55,56,57,58,59].

In order to further investigate the functional electrochemical performance of CWFZ2, a two-electrode SSC was directly constructed using two CWFZ2 electrodes (Figure 6a). Figure 6b shows the CV curves of the CWFZ2//CWFZ2 SSC from 5 mV s^−1^ to 200 mV s^−1^. All of the CV curves appear similar to those of the rectangles. Furthermore, the GCD curves show similar isosceles triangle shapes at various current densities, indicating good reversibility and EDLC behavior (Figure 6c). Notably, the device exhibits a capacitance of 29.86 F g^−1^ at a current density of 0.5 A g^−1^. An EIS analysis of CWFZ2//CWFZ2 was performed at 0.01 Hz to 100 kHz (Figure 6d). As shown, the Nyquist plot of this device shows a near-vertical line at low frequencies, indicating capacitive behavior [50]. The resistance of the SCs can be obtained as 1.11 Ω through the intercept of the curve and the abscissa. Furthermore, the SSC shows a high energy density of 4.15 Wh kg^−1^ at a specific power of 113.64 W kg^−1^ at a current density of 0.5 A g^−1^ (Figure 6e), which is higher than that of some the reported carbon-based devices [51,52,53,54,55,56,57,58,59]. At a current density of 10 A g^−1^, the specific power increases to 2272.73 W kg^−1^, and the specific energy remains at 2.46 W h kg^−1^. Although the power density has increased by a factor of ~20, the energy density has only dropped by 40.7%. The results show that CWFZ2 has great application potential in high-rate energy storage devices. The electrochemical cyclability with high retention (81.21%) after 10,000 cycles demonstrates the high reversibility and stable electrochemical behavior of the CWFZ2-based SC (Figure 6f).

## 3. Materials and Methods

### 3.1. Materials

WF was obtained from poplar wood through thermal grinding. The 2-methylimidazole (2-MeIm), Zn(NO_3_)_2_·6H_2_O, methanol, ethanol absolute, potassium hydroxide (KOH), polyvinylidene fluoride (PVDF), and acetylene black were purchased from Sinopharm Chemical Reagent Co., Ltd. (Shanghai, China). All the deionized water in the experiment was made by the laboratory.

### 3.2. Methods

#### 3.2.1. Preparation of WF@ZIF-8 Composite

First, WF was placed in a mixed solution of 10 mL methanol and 10 mL deionized water containing Zn(NO_3_)_2_·6H_2_O, and was continuously stirred for 30 min. Next, 10 mL methanol solution with 2-MeIm was added dropwise into the solution, and the mixture was continuously stirred for 2 h. Finally, the above materials were washed with methanol solution and deionized water and dried overnight at 70 °C in an oven to obtain the WF@ZIF-8 composite (WFZs). A series of WFZs denoted as WFZ1, WFZ2, and WFZ3 were prepared by subjecting the initial mass of wood fibers and the precursor of ZIF-8. The initial mass of components is provided in detail in Table 1.

#### 3.2.2. Preparation of WF@ZIF-8 Carbon Fibers

The WF@ZIF-8 carbon fibers (CWFZs) were prepared by placing the WF@ZIF-8 composite in a tubular furnace (OTF-1200X, Hefei Kejing Material Technology Co., Ltd., Hefei, China), which was subsequently flushed with pure N_2_ at a flow rate of 150 mL min^−1^. The temperature was increased to 800 °C (heating rate of 5 °C min^−1^), and this was maintained for 2 h. Finally, the tube furnace was naturally cooled to 25 °C, and the CWFZs were removed. The CWFZs obtained by the carbonization of WFZ1, WFZ2, and WFZ3 were named CWFZ1, CWFZ2, and CWFZ3, respectively.

#### 3.2.3. Characterizations

The structure and morphology of the different samples were characterized using scanning electron microscopy (SEM, Zeiss Sigma 300, Jena, Germany), transmission electron microscopy (TEM, JEM-1400plus, Japan Electronics Co., Ltd, Japan), and energy-filtered TEM mapping. The sample compositions were analyzed using X-ray diffraction (XRD, Bruke D8 Advance, Lufkin, TX, USA) with a Cu Kα source (λ = 1.54 Å, a scanning rate of 5°/min, and a scanning range of 10~80°), Raman (Horiba Scientific LabRAM HR Evolution), Fourier transform infrared spectroscopy (FTIR, Thermo Scientific Nicolet iS5), and X-ray photoelectron spectroscopy (XPS, Escalab 250Xi). The SSA and pore size distribution of the CWFZs were analyzed using the Brunauer–Emmett–Teller (BET) method (QUADRASORB SI).

#### 3.2.4. Electrochemical Performances

The electrochemical performances of the CWFZs were measured in an electrochemical workstation (CHI660E) using a three-electrode system. The acetylene black, PVDF, and CWFZs were made into a slurry (mass ratio of 1:1:8) and then coated onto foamed nickel (1 × 1 cm^2^) as the working electrode (the active material loading is about 2.4 mg). The three-electrode system used a 6 M KOH electrolyte with an Ag/AgCl_2_ electrode and a platinum plate as the reference and counter electrodes, respectively. Cyclic voltammetry (CV), galvanostatic charge/discharge (GCD), and electrochemical impedance spectroscopy (EIS) were conducted in an electrochemical workstation. From the GCD curves, the specific capacitance of the CWFZs was calculated using Formula (1),

*C* = (*I*△*t*)/(*m*△*V*), (1)

where △*t* is the discharge time, *I* is the current, △*V* is the working voltage, and *m* is the loading mass of the CWFZs. A simple SSC was assembled using two CWFZ2 electrodes and a separator (cellulose paper). The performance of the SSC was evaluated using a two-electrode system in an electrochemical workstation.

## 4. Conclusions

We have successfully developed an efficient method to obtain biomass carbon fiber electrode materials with excellent electrochemical performance. Among the carbon fiber electrode materials, the carbon of the wood fibers builds a conductive network for electron transport, and ZIF-8 provides a nitrogen source to improve the energy storage performance of the carbon material. The as-prepared CWFZ2 composite has a specific surface area of 593.52 m^2^ g^−1^, exhibits a high specific capacitance of 270.74 F g^−1^ in alkaline electrolyte, and exhibits an excellent capacitance retention of 98.4% after 10,000 cycles. The obtained SSCs show a high power density of 2272.73 W kg^−1^ at an energy density of 2.46 Wh kg^−1^. This work provides an efficient way to synthesize hierarchically porous N-doped carbon materials for high-performance supercapacitors.

## Figures and Tables

**Table 1:** Different initial mole content of ZIF-8 in the precursor.

Sample	Wood Fibers	Zn(NO_3_)_2_·6H_2_O	2-MeIm
WFZ1	0.5 g	0.5 g	0.5 g
WFZ2	0.5 g	0.5 g	1 g
WFZ3	1 g	0.5 g	1 g

## Data Availability

Not applicable.

## Sample Availability

Samples are available from the authors upon request.
